# Identification of a Novel Solinvivirus with Nuclear Localization Associated with Mass Mortalities in Cultured Whiteleg Shrimp (*Penaeus vannamei*)

**DOI:** 10.3390/v14102220

**Published:** 2022-10-09

**Authors:** Roberto Cruz-Flores, Thales P.D. Andrade, Hung N. Mai, Rod Russel R. Alenton, Arun K. Dhar

**Affiliations:** 1 Centro de Investigación Científica y de Educación Superior de Ensenada (CICESE), Carretera Ensenada-Tijuana No. 3918, Zona Playitas, Ensenada 22860, Baja California, Mexico; 2 Aquaculture Pathology Laboratory, School of Animal & Comparative Biomedical Sciences, The University of Arizona, Tucson, AZ 85721, USA; 3 Laboratório de Diagnóstico de Enfermidades de Crustáceos, Universidade Estadual do Maranhão, Cidade Universitária Paulo VI, 1000 Tirirical, São Luis 65055-970, MA, Brazil

**Keywords:** *Penaeus vannamei* solinvivirus, PvSV, shrimp, *Solinviviridae*

## Abstract

The emergence and spread of disease-causing viruses in shrimp aquaculture is not uncommon. Since 2016, unusual mortalities have been affecting the Brazilian shrimp industry and we have associated these unusual mortalities with a novel variant of infectious myonecrosis virus (IMNV). The transcriptome analysis of these diseased shrimp showed an additional divergent viral sequence that we have assigned to the family *Solinviviridae*. The novel virus has been tentatively termed *Penaeus vannamei* solinvivirus (PvSV) (GenBank accession: OP265432). The full-length genome of the PvSV is 10.44 kb (excluding the poly A tail) and codes for a polyprotein of 3326 aa. Five conserved domains coding for a helicase, RdRp, calicivirus coat protein, G-patch and tegument protein were identified. The genome organization of the PvSV is similar to other (*Nylan deria fulva* virus 1) solinvivirus. A unique feature of this virus that differs from other members of the *Solinviviridae* is the presence of putative nuclear localization signals. The tissue tropism of this virus is wide, infecting cells of the hepatopancreas, gastrointestinal tract, lymphoid organ and muscle tissue. Another unique feature is that it is the only RNA virus of penaeid shrimp that shows a nuclear localization by in situ hybridization. The PvSV has a wide distribution in Brazil and has been found in the states of Maranhão State (Perizes de Baixo), Piaui State (Mexeriqueira), Ceará State (Camocim, Jaguaruana, Aracati and Alto Santo) and Pará State where it has been detected in coinfections with IMNV. The diagnostic methods developed here (real-time RT-PCR and in situ hybridization) are effective for the detection of the pathogen and should be employed to limit its spread. Furthermore, the identification of the PvSV shows the increasing host range of the relatively new family *Solinviviridae*.

## 1. Introduction

As long as the culture of shrimp remains one of the most profitable sectors in the animal food production industry, the expansion of shrimp culture to new regions and the implementation of innovative culture techniques will continue. Without a doubt, this entails the movement of particular shrimp stocks across regions, countries and continents. The emergence and trans-boundary distribution of pathogenic agents associated with penaeid shrimp production is well documented [1] and is directly related to the movement of brood stock and shrimp by-products [2]. Disease emergence and spread in the shrimp industry corresponds to poor industry practices and have resulted in the cosmopolite distribution of every major pathogen that has ever affected the shrimp industry [1,3]. 

Viral diseases have been, without question, one of the biggest threats to the sustainability of shrimp aquaculture. Pathogens such as infectious hypodermal and hematopoietic necrosis virus (IHHNV), white spot syndrome virus (WSSV), Taura syndrome virus (TSV), yellow head virus (YHV) and infectious myonecrosis virus (IMNV) have caused major panzootics that had a global impact on shrimp prices and the livelihood of many people who directly and indirectly depend on shrimp aquaculture [4,5,6,7,8]. More recently, it has been evidenced that a particular IMNV strain causing unusual mortalities in Brazil could have been due to the movement of animals from Indonesia [9]. In addition, these same samples from Brazil exhibited the presence of another unique viral sequence corresponding to a novel virus that we initially proposed to belong to the family *Caliciviridae* [10], but we have now reclassified to be a member of the *Solinviviridae.* The presence of this novel virus in cultured shrimp from Brazil helps to explain the unusual mortalities that have been occurring since 2016 and shows the emergence of a novel pathogen that warrants direct monitoring to avoid its spread to other shrimp farming regions. 

The family *Solinviviridae* consists of picorna/calici-like viruses with a non-segmented linear positive-sense RNA genomes of 10–11 kb [11]. Unlike other viruses, their capsid proteins are encoded toward the 3′-end of the genome where they can be expressed both from a sub-genomic RNA and as an extension of replication [11]. Members of this family form icosahedral particles with a diameter of 26–30 nm and appear to have projections [12,13]. Currently, the family *Solinviviridae* includes two genera *Invictavirus* and *Nyfulvavirus* and are part of a large diverse group of arthropod-infecting viruses within the picorna/calici-like virus group of viruses [11]. Currently, no formal genus delineation criteria exist for this family. 

The *Solinviviridae* contain viral species that are known pathogens of ants and infect the midgut epithelium [14]. Some of these viruses cause chronic infections and low mortality that are restrained to one type (midgut epithelium) of tissue while the more virulent species causes systemic infections and acute mortality [12,14]. While the best studied species of solinivivirus are found in ants (*Solenopsis invicta virus* 3 and *Nylan deria fulva virus* 1), recent studies on the invertebrate RNA virosphere have shown that related unclassified virus sequences are present from other insects and arthropods [11,15]. In this study, we report the genomic characterization of a novel solinivivirus in the most important cultured shrimp species, *Penaeus vannamei,* and the diagnostic methodologies (qPCR and ISH) for its detection. We have provisionally named this virus “*Penaeus vannamei* solinvivirus” (PvSV).

## 2. Material and Methods

### 2.1. Sample Origin

The diseased *P. vannamei* examined in this study were collected from 2016 to 2019 from affected ponds located in the Brazilian northeast region including Maranhão State (Perizes de Baixo), Piaui State (Mexeriqueira), Ceará State (Camocim, Jaguaruana, Aracati and Alto Santo) and Pará State. The average weights of the diseased juvenile shrimp in the affected ponds were approximately 3.0 gm. Five to six shrimp were collected from each farm and screened by a real-time RT-PCR analysis, H&E histopathology and ISH. RNA isolation was performed using approximately 25 mg of the minced muscle and pleopods of the presumably affected shrimp. The Tissue LEV Total RNA Purification Kit (#AS1220, Promega, Madison, WI, USA) along with an automated DNA/RNA extraction system (Maxwell^®^ MDX Promega, USA) was used following the manufacturer’s recommendations. One-step real-time RT-PCR was carried out following the protocols described by Andrade et al. [16]. Prior to the real-time assay, extracted RNA was boiled at 100 °C for 3 min to denature dsRNA, then placed on ice and finally subjected to real-time RT-PCR analysis using GoTaq^®^ Probe 1-Step RT-qPCR system (PROMEGA, Madison, WI, USA). Each sample was analyzed in duplicate using a real-time PCR ViiA^7^ thermocycler (Applied Biosystems, Foster city, CA, USA).

### 2.2. Next Generation Sequencing

A sample 8-Br was sent to the Aquaculture Pathology Laboratory (APL) of the University of Arizona for pathogen screening. This sample had previously tested negative for all know viral, bacterial and fungal pathogens of cultured shrimp. The RNA from this sample was sent for RNA-Seq at OmegaBioservices, Norcross, GA, USA. Library preparation was carried out using a TruSeq Stranded Total RNA Library Prep (Illumina^®^, San Diego, CA, USA). The samples were sequenced using an Illumina HiSeq 2500 System (PE 2X150PE).

### 2.3. Bioinformatic Analysis

The 40,904,762 pair-end reads from sample 8-Br were checked for quality and trimmed before being mapped to the *P. vannamei* breed Keihai No.1 RefSeq Genome sequencing assembly (PRJNA508983) to remove the host reads using the Geneious Prime mapper with the default parameters (Biomatters) [17]. Duplicate reads were removed using Dedupe in Geneious Prime before mapping. The unmapped reads were de novo assembled using the Geneious prime assembler with the default parameters. The contigs and Open Reading Frames were analyzed by BLASTN and BLASTP search tools [18]. Motif search was performed using the Motif Search and Prosite Expasy to identify conserved domains in the viral sequence. Nuclear localization signals (NLSs) were predicted using NLStradamus using the 2-state HMM dynamic model with a prediction cutoff of 0.9 [19].

### 2.4. Phylogenetic Analysis

The RdRp domain of the PvSV from Brazil was compared with other viruses from the order *Picornavirales*. Sequences from the families *Caliciviridae* (AHX24377, AAL99277 and AYF53102), *Dicistroviridae* (NP620562, AAF80998 and AF277675), *Iflaviridae* (NP_853560, NP_049374, NP_277061 and NP_620559), *Marnaviridae* (NP_944776), *Picornaviridae* (NP_740737, BAA31356 and NP_740478), *Polycipiviridae* (APG774337, ASK12200 and ASK12194), *Secoviridae* (NP_734447, NP_734463, YP_054443, NP_730557, YP_001039627 and YP_081454) and *Solinviridae* (AAZ78308, ANQ44728 and ACO37271) were used in the analysis. The sequences were aligned using the Geneious Aligner and the phylogenetic analysis was conducted using MEGAX [17,20]. A phylogenetic tree was constructed using the neighbor-joining method [20]. The bootstrap consensus tree was inferred from 1000 replicates.

### 2.5. Primer Design and Cloning of the PvSV Genomic Fragment

Using the generated PvSV genome from the NGS data set, a primer pair and a probe were designed (Table 1). Primers and probes were designed using Geneious Prime and were tested for specificity using Primer-Blast [17,21]. The primer and probe binding regions were selected by the Geneious Prime Primer Design Algorithm considering the optimal fragment size rage of 100–220 bp and an annealing temperature of 60 °C. Primer-Blast analyses indicated that the primers were specific to PvSV. The TaqMan probe was synthesized and labeled with 6-carboxyfluorescein (FAM) on the 5′ end and N, N, N′,N′-Tetramethyl-6-carboxyrhodamine TAMRA on the 3′-end. For the assays, TaqMan Fast Virus 1-Step Master Mix (Applied Biosystems™, Foster city, CA, USA) was used, the final concentration for each primer was 0.5 µM and 0.1 µM for the TaqMan probe at a final volume of 10 µL. The qPCR profile was 20 s at 95 °C followed by 40 cycles of 1 s at 95 °C and 20 s at 60 °C.

The cDNA fragment of 133 bp was amplified from the original sample 8-Br. and cloned into the pDrive Cloning Vector (QIAGEN^®^, Hilden, Germany) to be used as a positive control. The cloned plasmid was designated as PvSV-8-Br. Plasmid DNA, was purified using QIAprep^®^ Spin Miniprep Kit. The sequence of the PvSV-8-Br fragment was verified by sequencing at the sequencing facility of The University of Arizona, Tucson, AZ, USA.

### 2.6. Histopathology

All shrimp samples were fixed with Davidson’s fixative (330 mL 95% ethanol, 220 mL 100% formalin, 115 mL glacial acetic acid, 335 mL distilled water, pH~3.0–4.0) following a standard procedure [22]. After fixation, Davidson’s fixative was removed and replaced with an equal volume of 70% ethanol. For histological processing, samples were washed in a series of alcohol/xylene solutions, embedded in paraffin, sectioned at 5 µm and stained with H&E following a standard procedure [22]. Histological slides were examined using a bright field light microscope. The severity of the infection was graded based on a semi-quantitative scale that ranges from Grade 0 to Grade 4 following a previous publication [23].

### 2.7. In Situ Hybridization

All sections were processed as previously described (histopathology). However, for ISH the sections were dried onto positively charged microscopic slides and ISH was carried out following the protocols described by Cruz-Flores et al. [24], with an equal volume mixture of the same primers (3136 F and 3268 R) for qPCR and the probe (Probe 3159). These primers and probe were tailed at 3′-end with digoxigenin-11-dUTP (Sigma-Aldrich™, St. Louis, MO, USA). A paraffin block containing previously-known PvSV affected shrimp was used as positive control. As a negative control, paraffin blocks of specific pathogen free shrimp were used. Slides were examined by light microscopy for the presence or absence PvSV hybridizing with the DNA probe, and the slides that showed blue to purple precipitates were considered positive.

## 3. Results

### 3.1. Penaeus vannamei Solinvivirus Genome Organization and Sequence Analyses

**Figure 1:**
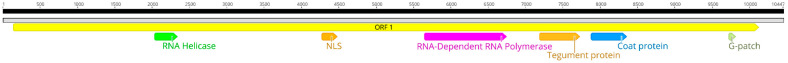
Genome organization of the *Penaeus vannamei* solinvivirus (PvSV). The genome consists of one large ORF with five identified conserved domains including an RNA helicase (green), Nuclear localization signal (orange), RNA-dependent RNA polymerase (pink), tegument protein (orange), Calicivirus coat protein (blue) and G-patch (light green).

The full-length genome of PvSV is 10.44 kb (excluding the poly A tail) and presented a mean coverage of 9853. We identified one large open reading frame (ORF) (9981 nt) that codes for a polyprotein of 3326 aa with similarity to Picornavirales sp. and Riboviria sp (Table 2). Motif search identified four conserved domains coding for a Helicase, RNA-dependent RNA polymerase (RdRp), calicivirus coat protein, G-patch and tegument protein (Figure 1). The genome organization is similar to other (*Nylan deria fulva virus* 1) solinvivirus displaying one large ORF. The nuclear localization signal (NLS) analysis indicates that there is a putative NLS in the polyprotein around 4268–4474aa. The genome sequence of PvSV was uploaded to GenBank under accession number OP265432.

The sequence analysis of ORF1 showed an identity of 93.84% to the Wenzhou shrimp virus 8 (NC_032852.1), an unclassified Riboviria. Furthermore, amino acid (aa) sequence analysis of the helicase and RdRp conserved domains showed a 100% and 99.22% similarity to the unclassified Wenzhou shrimp virus 8 hypothetical protein (YP_099336733.1), as seen in Table 2.

### 3.2. Phylogeny

**Figure 2:**
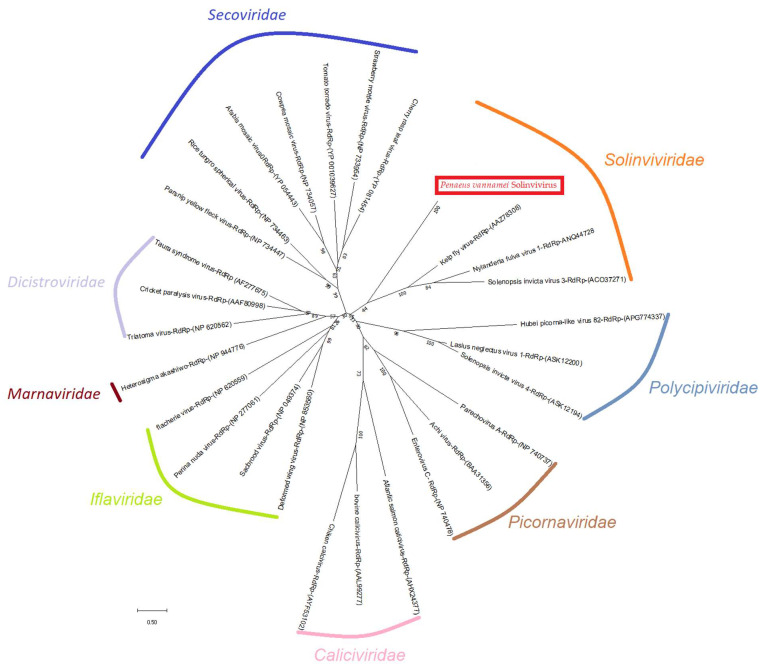
The evolutionary history of *Penaeus vannamei* solinvivirus (PvSV) was inferred using the Neighbor-Joining method. The figure shows the PvSV (red box) clusters with the family *Solinviviridae* and is divergent from the rest of the viruses in this family. The optimal tree with the sum of branch length = 16.70591706 is shown. The percentage of replicate trees in which the associated taxa clustered together in the bootstrap test (1000 replicates) are shown next to the branches. The tree is drawn to scale, with branch lengths in the same units as those of the evolutionary distances used to infer the phylogenetic tree. The evolutionary distances were computed using the Poisson correction method and are in the units of the number of amino acid substitutions per site. This analysis involved 29 amino acid sequences. All ambiguous positions were removed for each sequence pair (pairwise deletion option). There was a total of 610 positions in the final dataset.

The phylogenetic inferences using the neighbor-joining method for the RdRp indicated that the novel virus clusters with the family *Solinviviridae* possibly represented a new genus within this family (Figure 2).

### 3.3. Penaeus vannamei Solinvivirus Distribution in Brazil

The primers designed for the PvSV amplify a fragment of 133 nt of the viral genome. The cloned fragment was sequenced using the Sanger method and it showed an identity of 99.9% to PvSV complete genome sequence. This plasmid was subsequently used as a positive control for qPCR detection assays.

The PvSV is widely distributed throughout Brazilian farms and is present in the states of Maranhão State (Perizes de Baixo), Piaui State (Mexeriqueira), Ceará State (Camocim, Jaguaruana, Aracati and Alto Santo) and Pará State. The total number of positive cases from 2016 to 2019 was recorded using the PvSV real-time RT-PCR assay. In this study, of the 13 selected suspected cases, PvSV was detected in 11 cases (84%) (Table 3).

### 3.4. Histopathology and In Situ Hybridization

**Figure 3:**
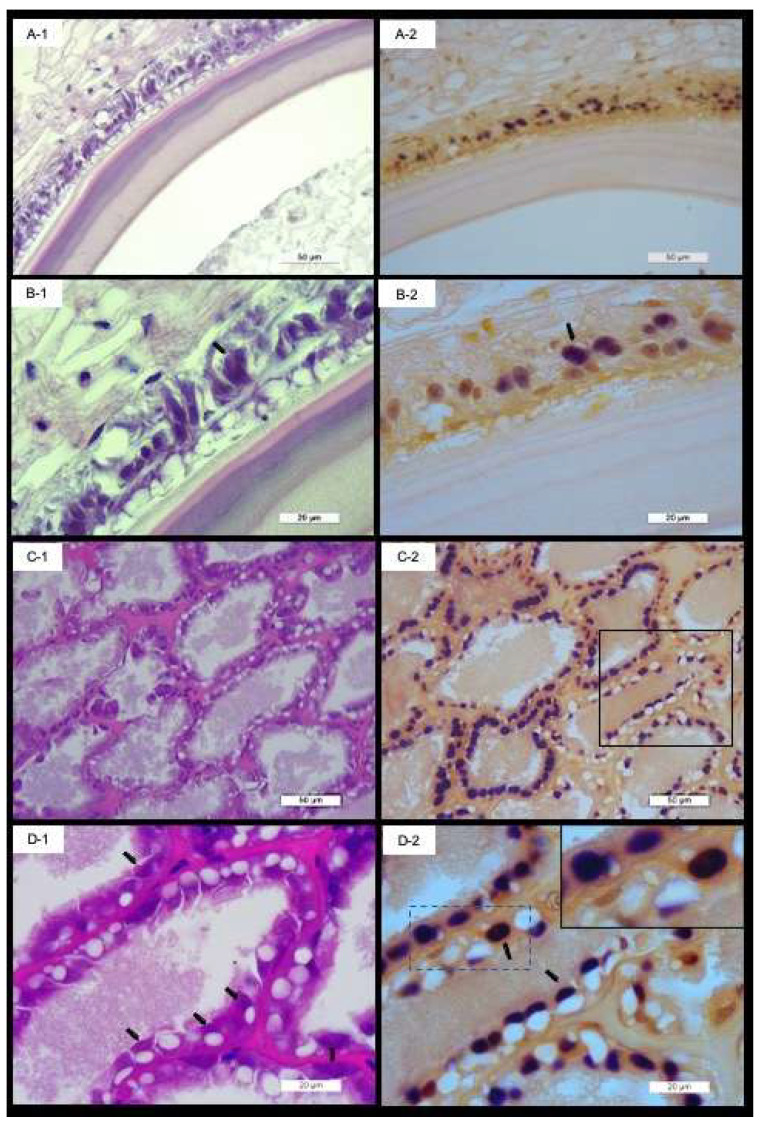
Hematoxylin and eosin-stained sections and in situ hybridization of parallel sections of the gut and hepatopancreas of *Penaeus vannamei* infected with *Penaeus vannamei* solinvivirus (PvSV). (**A-1**–**D-1**) Basophilic and eosinophilic viral inclusions in the epithelial cells of the gut, stomach and hepatopancreas tubules of *P. vannamei* with PvSV; these are shown by the black arrows. The positive signal by the DIG-labeled probes is observed in the nucleus of the epithelial cells of the gut and the hepatopancreas (**A-2**–**D-2**). The box with solid line in Panel **C-2** is shown in Panel **D-2**, and the box in dotted line is shown in the right-hand corner in Panel **D-2**.

**Figure 4:**
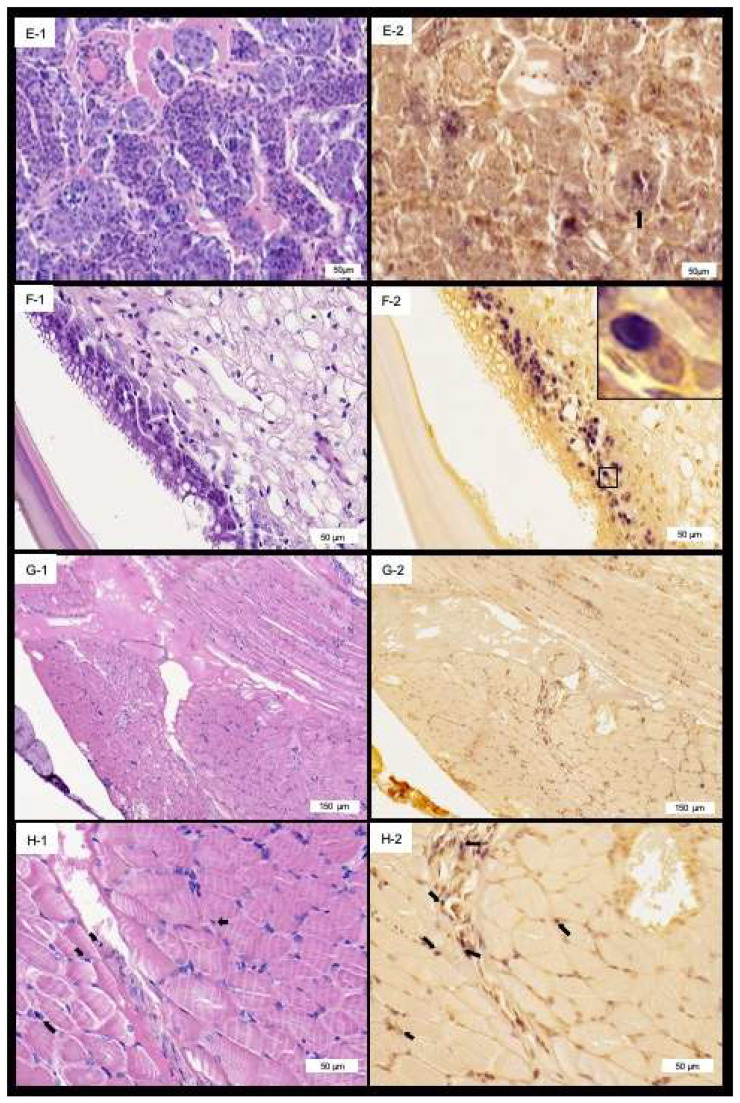
Hematoxylin and eosin-stained sections and in situ hybridization of parallel sections of *Penaeus vannamei* tissue infected with the *Penaeus vannamei* solinvivirus (PvSV). (**E-1**) Lymphoid organ spheroids in diseased shrimp. (**E-2**) Lymphoid organ spheroids showing both a cytoplasmic and nuclear reaction to the PvSV-specific probe. (**F-2**) Gut epithelia showing a large number of intranuclear inclusions with a positive reaction to the PvSV probe, no apparent abnormalities are observed in the infected nuclei (**F-1**). (**G-1**–**H-2**) Low and high magnification of the muscle tissue also showing a positive reaction in the nuclei. A magnified view of the box with solid line in Panel **F-2** is shown in the right-hand corner of the panel.

The PvSV infects cells of the gastrointestinal tract (Figure 3). The signal of the DIG-labeled probes was observed in the nucleus of the epithelial cells of the hepatopancreas tubules, the epithelial cells of the gut and the stomach (Figure 3 and Figure 4F-1,F-2). In the lymphoid organ, the signal of the DIG-labeled probe was observed in both the cytoplasm and the nucleus (Figure 4E-1,E-2). Similarly, the signal was observed in the nucleus in the cells of the striated muscle (Figure 4G-1,G-2,H-1,H-2). No reaction to the PvSV-specific probe was observed in the SPF shrimp tissue (Figure S1).

## 4. Discussion

Since 2016, unusual mortalities that progressed noticeably fast and resulted in a higher cumulative mortality (up to 80%) have been recorded in the states of Pará, Maranhão, Piaui, Ceará, Rio Grande do Norte, Alagoas, Sergipe and Bahia in Brazil [9]. As we previously mentioned, we found that these mortalities are associated with a novel strain of IMNV [9]. In addition, during the transcriptome screening of these IMNV-infected shrimp, we identified an additional divergent viral sequence that corresponds to a novel member of the *Solinviviridae* that we have tentatively named PvSV. The identification of PvSV in Brazilian shrimp experiencing unusual mortalities in co-infection with IMNV raises question regarding whether there is any synergistic interaction between the two viruses, contributing to a more rapidly progressing disease and higher mortalities observed in different Brazilian states.

The transboundary movement of brood stock and post larvae between shrimp farming nations remains a main reason for which all the major shrimp pathogens have a cosmopolitan distribution [9,25,26]. A novel strain of IMNV recently identified in Brazil possibly originated in Asia and was recently reintroduced in Brazil [9]. The PvSV shows a high similarity to Wenzhou shrimp virus 8 which was originally detected in Asia in 2016 and might represent a divergent strain of this virus [15]. Additional viral sequences from Thailand [27], China [28] and Australia [29] have been found to also present a high similarity to the Wenzhou shrimp virus 8 and could therefore also represent viruses related to PvSV. This raises questions regarding whether PvSV was introduced in Brazil via the movement of animals from the mentioned shrimp farming nations. However, whether a newly emerged strain of IMNV and PvSV were introduced simultaneously or in different introduction events remains unknown.

The *Solinviviridae* is a family of viruses that infects ants, but related viruses infect a large variety of insects and other arthropods [11,12,13,30]. These viruses are related to the *Caliciviridae* and *Picornaviridae,* also possessing linear, non-segmented, positive-sense RNA genomes of ~10–11 Kb [11]. However, the *Solinviviridae* differs from the *Caliciviridae* in terms of genome size and ORF organization. The *Caliciviridae* have shorter genomes, such as 6.4–8.5 kb genomes with two to three ORFs [31]. We had initially assigned the PvSV to the family *Caliciviridae* considering the calicivirus coat protein motif and limited phylogenetic analysis [10]. However, upon additional phylogenetic analysis and the taxonomic assignment of the Wenzhou shrimp virus 8 to the family *Solinviviridae* in the International Committee on Taxonomy of Viruses chapter for the *Solinviviridae,* we have concluded that the novel shrimp virus from Brazil is a member of this family [32]. The taxonomical affiliation of the *Penaeus vannamei* picornavirus (PvPV) [28] should be further evaluated using the putative RdRp region to clarify its location in the order *Picornavirales*. The *Solinviviridae* is a relatively new family with most members of the family remaining unclassified. Only two members have been assigned to date, namely the *Invictavirus* and *Nyfulvavirus* [11,13,33]. The PvSV represents a novel genus within the family and is the first solinvivirus known to infect penaeid shrimp. The novel shrimp virus presents the typical size and genome organization for the family but shows an additional feature, namely the presence of the NLS. The NLS have not been described for the *Solinviviridae* and this is the first evidence of nuclear localization for this virus group confirmed by in situ hybridization. The tissue tropism of PvSV (replicates in the hepatopancreas) is similar to other solinvivirus that infect the midgut epithelia of insects [12,13,30] but differs in the fact that it affects muscle tissue. The PvSV infection is enteric and systemic like the *Solenopsis invicta virus* 3 which affects all tissues of the host [12].

The affinity of PvSV to the hepatopancreas and its effect on the tissues makes this organ more susceptible to chronic bacterial infections. The tissue tropism of the PvSV is almost identical to the Wenzhou shrimp virus 8-like virus detected in Thailand [27]. Due to the signs that are displayed by infected shrimp that may be associated with a bacterial etiology, Brazilian farmers usually suspected and tested for other pathogens that cause enteric diseases such as necrotizing hepatopancreatitis due to *Hepatobacter penaeid,* acute hepatopancreatic necrosis disease (Vp_AHPND_), hepatopancreatic microsporidiosis caused by *Enterocytozoon hepatopenaei* (EHP) and septic hepatopancreatic necrosis due to vibriosis (SHPN). However, it is clear from the results of this study and our previous studies [9] that the coinfection of PvSV and IMNV are a likely cause for the unusual mortalities that are currently affecting Brazilian shrimp farming. Further studies are required to demonstrate Rivers’ postulate to determine the infectivity of PvSV and evaluate its role in the unusual mortalities occurring in shrimp farms in Brazil.

The PvSV is widely distributed in Brazil, affecting seven states in the northeast region, and it has been detected in both juveniles and post-larvae. It is possible that the PvSV has a much wider distribution in Latin America and future detection studies are required to determine its prevalence and its possible interactions with other shrimp pathogens. Coincidentally, the distribution of this pathogen encompasses an area where most of the national Brazilian shrimp production areas are located, and this region is currently experiencing a toll on production. It is tempting to speculate that a cumulative effect of a dual infection of IMNV and PvSV is contributing to this loss. The real-time RT-PCR and ISH methodologies described here will allow for the testing of brood stock and post-larvae to limit the further spread of the pathogen and will help mitigate its negative effects on Brazilian shrimp farming and other shrimp farming nations. Furthermore, the genetic characterization and taxonomic assignment of the PvSV sheds light on the genetic diversity and host range of the family *Solinviviridae*.

## Figures and Tables

**Table 1:** Primers and probes designed for the specific detection of the *Penaeus vannamei* solinvivirus (PvSV) by real-time RT-PCR.

Primer/Probe Name	Primer Sequence (5′ to 3′)	Product Size (nt)
3136 F (Set 1)	TACGCCACGAACGAGAACAA	133
3268 R (Set 1)	GGACAGCGACAAAGACGAGA	
Probe 3159 (Set 1)	[FAM]CGTCGTGACTACTCTCACCG [TAM]	

**Table 2:** Amino acid sequence similarity of ORF1, Helicase and RNA-dependent RNA polymerase of *Penaeus vannamei* solinvivirus (PvSV) to other viruses from GenBank.

Virus	Accession	Query Cover (%)	E-Value	Percent Identity (%)
**Whole Genome**				
Wenzhou shrimp virus 8	KX883984.1	98	0.0	93.14
*Penaeus vannamei* picornavirus	UIU06302.1	75	0.0	94.67
*Penaeus vannamei* picornavirus	UIU06303.1	23	0.0	82.72
**Helicase**				
Hypothetical protein (Wenzhou shrimp virus 8)	YP_009336733.1	100	4 × 10^−63^	100.00
*Penaeus vannamei* picornavirus	UIU06302.1	75	5 × 10^−63^	100.00
Hypothetical protein (*Diabrotica virgifera virgifera* virus 3)	YP_009352234.1	95	2 × 10^−12^	35.05
**RNA-dependent RNA polymerase**				
Hypothetical protein (Wenzhou shrimp virus 8)	YP_009336733.1	100	0.0	99.22
*Penaeus vannamei* picornavirus	UIU06302.1	100	0.0	99.18
Hypothetical protein (Hubei picorna-like virus 49)	YP_009336567.1	89	6 × 10^−7^	34.45

**Table 3:** Detection of IMNV and PvSV by real-time RT-PCR in *Penaeus vannamei* collected from shrimp farms experiencing disease outbreaks. These farms are located in four different States in Brazil. ND = Not detected.

Year	State	Location	Sample	IMNV Ct Value	PvSV Ct Value
2016	Ceará	Aracati CE01	2016.1	29	35
2017	Ceará	Alto Santo CE02	2017.5	32	24
	Ceará	Jaguaruana CE03	2017.16	ND	20
2018	Piaui	Mexeriqueira PI1	2018.04	24	37
	Ceará	Alto Santo CE04	2018.06	15	ND
	Piaui	Mexeriqueira PI2	2018.07	27	34
	Ceará	Camocim CE21	2018.08	18	28
	Ceará	Camocim CE05	2018.21	28	38
	Maranhão	Perizes de baixo MA01	2018.42	10	36
2019	Ceará	Jaguaruana CE06	2019.14	ND	ND
	Ceará	Jaguaruana CE14	2019.14	31	27
	Pará	PA01	2019.16	18	33
	Ceará	Aracati CE07	2019.42	32	34

## Data Availability

The raw nucleotide sequence data reported in this manuscript are available in the NCBI Sequence Read Archive (SRA) databases under the BioProject ID: PRJNA871275, SubmissionID (SUB11953069) (https://www.ncbi.nlm.nih.gov/bioproject/871275 (accessed on 20 September 2022)).

## Supplementary Materials

The following are available online at https://www.mdpi.com/article/10.3390/v14102220/s1.

## Abbreviations

PvSV	*Penaeus vannamei* solinvivirus
IMNV	Infectious myonecrosis virus
ISH	In situ hybridization
