# Supplementary material for: Identification of a Novel Solinvivirus with Nuclear Localization Associated with Mass Mortalities in Cultured Whiteleg Shrimp (Penaeus vannamei)
Source: Viruses. 2022 Oct 9;14(10):2220. doi: 10.3390/v14102220 (PMC9610163; doi:10.3390/v14102220)
Supplement: Supplementary file 1 [file viruses-14-02220-s001.zip › viruses-1909366-supplementary.pptx]

## Slide 1
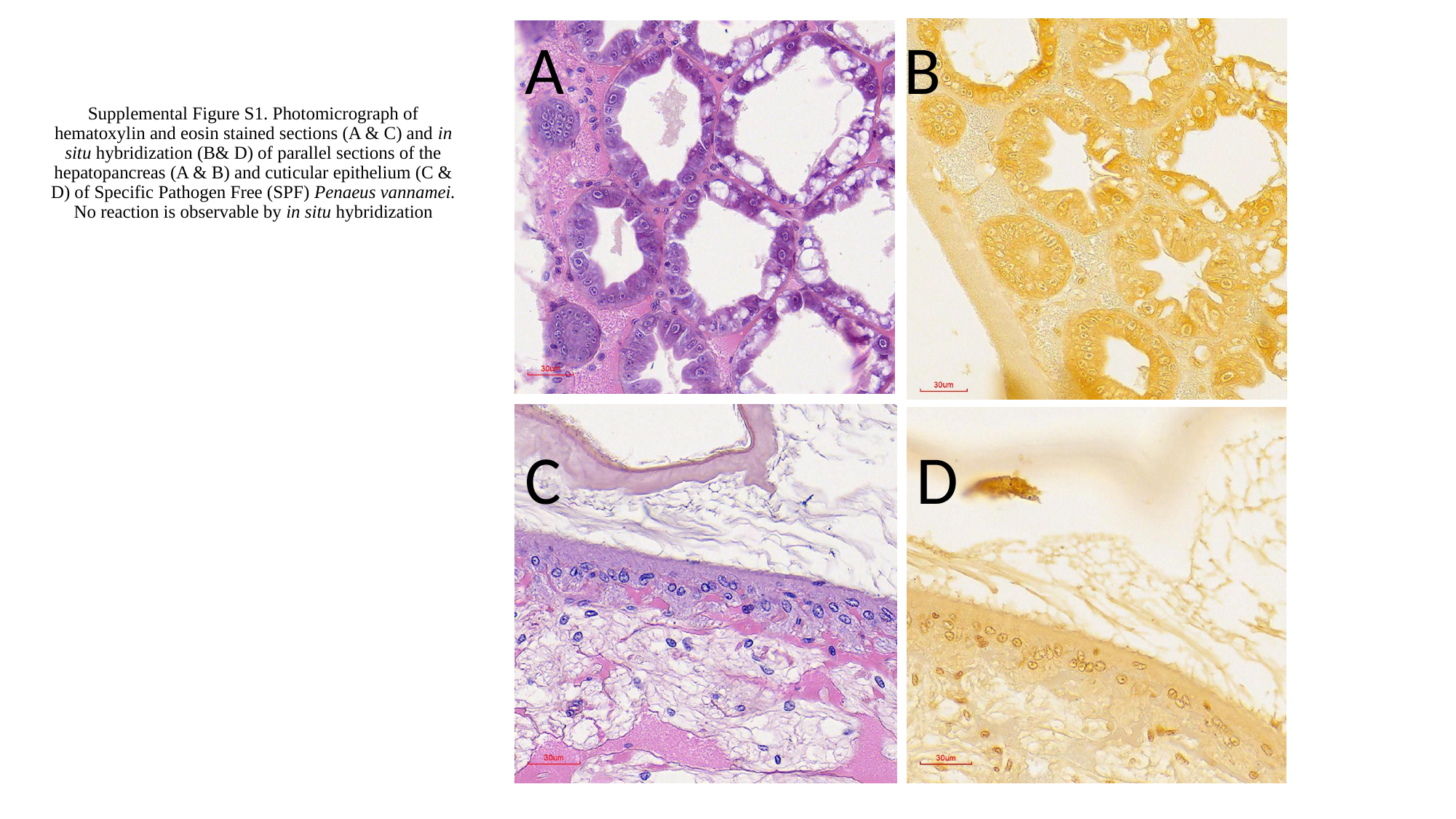

A B
C D
# Supplemental Figure S1. Photomicrograph of hematoxylin and eosin stained sections (A & C) and in situ hybridization (B& D) of parallel sections of the hepatopancreas (A & B) and cuticular epithelium (C & D) of Specific Pathogen Free (SPF) Penaeus vannamei. No reaction is observable by in situ hybridization
